# Release From Captivity Allows African Savannah Elephant Movement Patterns to Converge With Those of Wild and Rehabilitated Conspecifics

**DOI:** 10.1002/ece3.72597

**Published:** 2025-12-02

**Authors:** Murphy Tladi, Mike Murray‐Hudson, Andre Ganswindt, Emily Bennitt

**Affiliations:** ^1^ Okavango Research Institute University of Botswana Gaborone Botswana; ^2^ Mammal Research Institute University of Pretoria Pretoria South Africa

**Keywords:** *Loxodonta africana*, movement patterns, reintegration, rewilding, telemetry, translocation success

## Abstract

Rewilding captive animals is an important strategy for rehabilitating individuals and ecosystems. Comparing the behaviors of released animals to their wild counterparts enables the evaluation of their adaptive response to new environments, assuming that wild animals are better suited to natural conditions. We examined how movement patterns of captive African savannah elephants ( *Loxodonta africana* ) before and after soft release compared with movement patterns of other elephant groups, rehabilitated and wild elephants, in the western Okavango Delta, Botswana. We monitored 12 adult female elephants using GPS collars: six captive elephants, subjected to a three‐year phased soft release, two elephants released more than a decade earlier and four wild elephants. We quantified 30‐min diurnal and nocturnal distances, cumulative daily distances, daily displacement, and monthly home range sizes across seasonal flood cycles. We analyzed the effects of release, season, time of day, and elephant group on movement metrics, comparing captive elephants before and after release, and with rehabilitated and wild elephants. Before release, captive elephants moved longer diurnal and shorter nocturnal 30‐min distances, covered longer cumulative daily distances, and occupied smaller home ranges. After release, these metrics shifted, reducing differences with rehabilitated and wild elephants, although captive elephant home ranges remained significantly smaller. This suggests that captive elephants changed their movement patterns post‐release in response to environmental cues. However, even the movement patterns of rehabilitated elephants were not completely similar to those of wild elephants, likely due to sample size, individual variation, or effects of prior taming. These results highlight the critical importance of long‐term monitoring of animals since the movement patterns of released animals may take several years to converge with those of wild counterparts.

## Graphical abstract

African Savannah Elephants from captivity were rewilded and their movement patterns were compared with those of rehabilitated and wild elephants during the 3‐year soft release. The movement patterns of the captive elephants changed after release and were less different from most of the movement patterns of rehabilitated elephants and some of the movement patterns of wild elephants. The movement patterns of the captive elephants were affected by season after release just like rehabilitated and wild elephants.

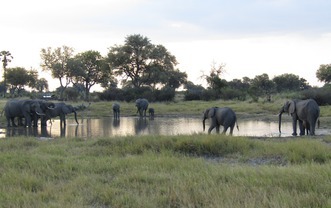

## 1 Introduction

The release of captive animals into the wild through translocation is essential for conservation efforts aimed at restoring populations, enhancing biodiversity, and the rehabilitation of individuals or ecosystems (IUCN/SSC 2013). The survival of translocated animals in new environments rests upon their ability to navigate and form spatial memories, which is critical for locating resources, avoiding threats, and establishing appropriate home ranges (Berger‐Tal and Saltz 2014). Therefore, assessing movement patterns of translocated animals is key to determining an animal's capacity to behaviorally adapt to unfamiliar environmental and social conditions (Owen‐Smith and Cain 2007). After translocation, some animals may exhibit homing behavior (Hinderle et al. 2015), travel long distances away from the release area (Mihoub et al. 2011) or become highly sedentary (Loonstra et al. 2023).

Movement patterns can be species dependent: mule deer ( *Odocoileus hemionus* ) and moose ( *Alces alces* ) occupy smaller home ranges than blue wildebeest ( *Connochaetes taurinus* ) and barren‐ground caribou ( *Rangifer tarandus granti* ) (Morrison et al. 2021). Different individuals may exhibit different movement adaptive responses, which might be affected by the history of the animals or the release strategy used (Shier and Swaisgood 2012). Captive animals, in particular, are disadvantaged due to restricted movement, altered diets, and lack of access to the natural environment in captivity (Tetzlaff et al. 2019), which can influence their susceptibility to predation, ability to locate limited resources, and response to environmental demands in the wild (Doyle et al. 2024; Chopra et al. 2025). Critical behaviors necessary for survival in the wild are likely to be affected by the duration of captivity and type of release used (De Milliano et al. 2016; Resende et al. 2021).

Animals in the wild are behaviorally well adapted to the ecological demands of their environment, optimizing their movement patterns to access resources such as water, food, and shelter (Loarie et al. 2009). When resource availability varies seasonally, wild animals show corresponding shifts in their movement dynamics, highlighting their behavioral adaptations to the demands posed by the environment (Egevang et al. 2010). For example, studies on African savannah elephants ( *Loxodonta africana* ) (Garstang et al. 2014), zebras ( *Equus quagga* ) (Sianga et al. 2025), and African buffalo ( *Syncerus caffer* ) (Naidoo et al. 2012), demonstrated variation in movement behaviors, including shifting locations and season‐dependent changes in size and habitat composition of their home ranges. These behaviorally adaptive movement strategies serve as a natural baseline against which the movements of rewilded animals can be compared, providing insights into the efficacy of release projects and the behavioral plasticity of released animals (Goldenberg et al. 2021). Therefore, understanding the movement patterns of wild animals provides a baseline to which those of released captive elephants can be compared.

Despite the recognized importance of movement behavior in translocation programs, there remains a knowledge gap regarding how captive animals adjust their movement dynamics during rewilding, particularly in comparison with their wild conspecifics and, in some cases, with rehabilitated animals in the same environment (Moro 2003; Pinter‐Wollman et al. 2009; Doden et al. 2022). The comparison could be used to advise on the required post‐release monitoring duration and assess rewilding outcomes (Chopra et al. 2025). For example, Moehrenschlager and Macdonald (2003) used the movement patterns of resident swift foxes ( *Vulpes velox* ) to determine the settlement phase in translocated conspecifics.

**FIGURE 1:**
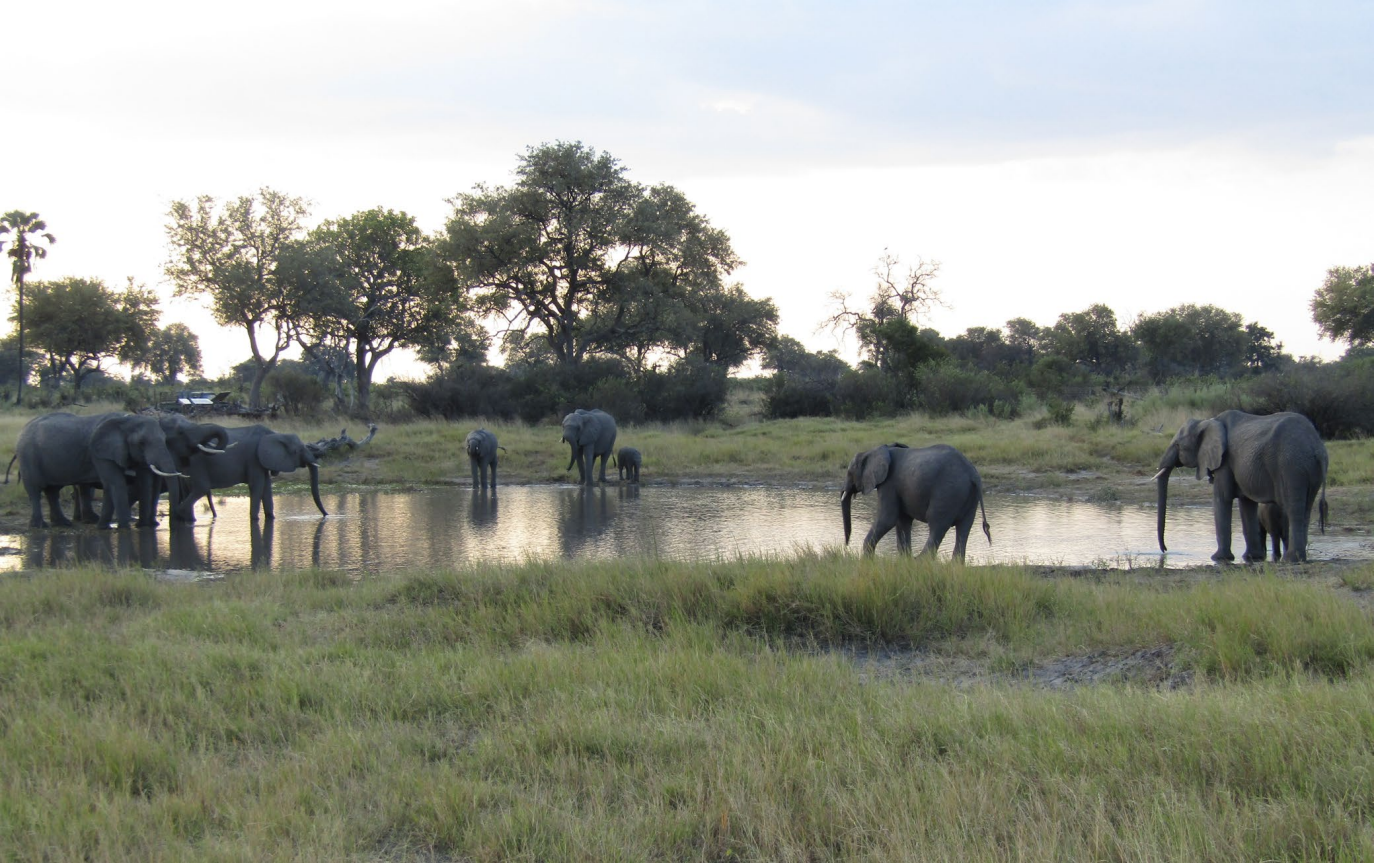
Captive elephants being herded back to the overnight enclosure (boma) in Abu Private Reserve, Okavango Delta, Botswana.

This study compared the seasonal movement patterns (diurnal and nocturnal 30‐min distances, cumulative daily distances, daily displacement, and monthly home range sizes) of captive African savannah elephants (Figure 1) in the western Okavango Delta before and after a soft release. The movement patterns were compared with those of rehabilitated (rewilded, formerly captive) and wild African savannah elephants within the same ecological context. We hypothesized that (i) before release, captive elephants would exhibit lower values of all movement metrics than after release due to confinement in controlled environments; (ii) before release, captive elephants would have significantly smaller values of all movement metrics than rehabilitated and wild elephants, which would not differ significantly from each other; (iii) after release, captive elephants' movement metric values would not be significantly different from rehabilitated and wild elephants; (iv) rehabilitated and wild elephant movement metrics would not be significantly different from each other; (v) all elephants would have lower 30‐min nocturnal than diurnal distances; (vi) before release, captive elephants' movement metrics would not be affected by season due to controlled conditions; (vii) for captive elephants after release, and for all other elephants, movement metrics would be significantly greater during the rainy season than in the early and late flood seasons. Findings from this study will provide critical insights into the rewilding process and inform the development of evidence‐based release strategies for captive elephants and other large mammals.

## 2 Methods

### 2.1 Study Area

**FIGURE 2:**
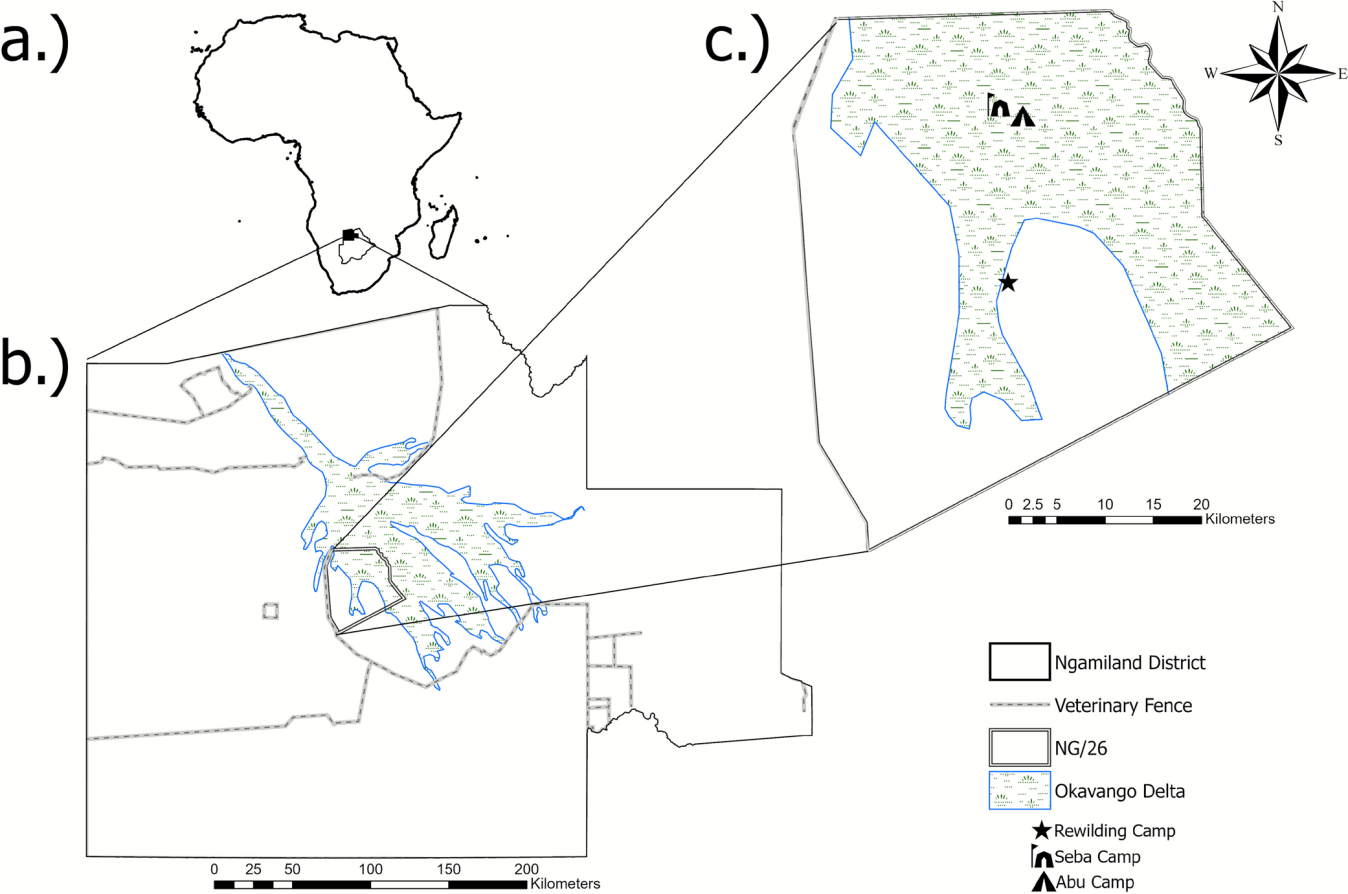
Map showing the position of the study area in relation to (a) Africa and Botswana, (b) Botswana Northwest district, and (c) the Abu Private Reserve. The elephants were translocated from Abu Camp to be soft released at the Rewilding Camp.

The Abu Private Reserve (APR, Controlled Hunting Area NG/26), Botswana, is located on the western side of the Okavango Delta alluvial fan (Figure 2). The Okavango Delta experiences a flood‐pulsed hydroperiod, whereby rains falling in Angola during the regional rainy season (October to April) fill river systems, leading to an annual flooding event in Botswana several months later (Ramberg et al. 2006). Seasons were defined according to water availability as rainy (December–March), early flood (April–July), when the Okavango Delta floodwaters rise, and late flood (August–November) when the Okavango Delta floods recede (Bennitt et al. 2015). The Okavango Delta is home to a variety of animals, including large herbivores such as African savannah elephants, hippopotamus ( *Hippopotamus amphibius* ), and Cape buffalo (*Syncerus caffer caffer*) (Ramberg et al. 2006; Moliner Cachazo et al. 2023). The vegetation in the area includes a mosaic of riparian trees, shrubs, grasses, and sedges featuring common species such as sycamore fig ( *Ficus sycomorus* ), fever berry ( *Croton megalobotrys* ), fan palm ( *Hyphaene petersiana* ), salt grass (*Sporobolus spicatus*), common finger grass ( *Digitaria eriantha* ), and hispid sedge (*Abildgaardia hispidula*) (Ellery et al. 1993; Sianga and Fynn 2021; Murray‐Hudson et al. 2014; Ramberg et al. 2006).

### 2.2 Study Animals

Historically, APR supported elephant‐back safaris on tamed and trained African savannah elephants. For ease of management, some of the trained elephants in APR were released from captivity as individuals or in batches (Evans et al. 2013a, 2013b). Elephant‐back safaris were halted in 2016, and all interactions between trained elephants and tourists ceased in 2019. The trained elephants were kept in a semi‐captive state where they were herded from overnight sleeping areas (bomas) in the morning and allowed to forage and interact with other elephants in the wild before they were returned to the secured sleeping bomas every night. The soft release of six adult female elephants (hereafter known as Captive elephants; Table 1) and their three offspring were initiated in 2021; three additional calves were born during the release, and one juvenile male left the group. Only adult females were collared in this study, as their offspring were too small to wear collars. No adult male elephants were in captivity during this study, having been released due to complications when in captivity (Evans et al. 2013a). One of the captive elephants died two years into the soft release due to old age, and the other was euthanized three months before the end of the soft release following an encounter that put humans at risk. Two adult female elephants had been hard released in 2003 and 2011 (hereafter known as Rehabilitated elephants) (Evans et al. 2013b); these individuals were often seen together as a group and visited Abu Camp regularly, so they were also fitted with collars.

**TABLE 1:** Data collection periods for captive, rehabilitated, and wild elephants in Abu Private Reserve, Okavango Delta, Botswana.

Elephant	Captivity level	GPS data collection start	GPS data collection end
Cathy	Captive	October 2021	November 2023
Lorato	Captive	October 2021	November 2024
Naledi	Captive	October 2021	August 2024
Paseka	Captive	October 2021	November 2024
Sirheni	Captive	October 2021	November 2024
Warona	Captive	October 2021	November 2024
Gika	Rehabilitated	December 2021	November 2024
Nandipa	Rehabilitated	December 2021	November 2024
Wild1	Wild	December 2021	November 2024
Wild2	Wild	December 2021	November 2024
Wild3	Wild	December 2021	April 2023
Wild4	Wild	June 2023	November 2024

To allow a comparison among wild elephants and those that had experienced captivity, three wild adult females (hereafter called wild elephants), representing three wild herds, were collared during the soft release process. The wild herds were found close to the release site and had similar herd sizes and demographic compositions to the captive elephant herd. One of the wild elephants had its collar mistakenly removed halfway through the study, and the collar was redeployed onto another wild elephant matriarch within a month. Wild elephant herds served as a baseline to which the movement patterns of captive and rehabilitated elephants were compared. In total, data were collected from twelve elephants, from these three elephant groups.

### 2.3 GPS Collars

The elephants were fitted with GPS‐enabled satellite collars (Vectronics Aerospace, Berlin, Germany) programmed to collect GPS fixes every 30 min; these data were downloaded using GPS Plus X software (Vectronics Aerospace, Berlin, Germany). Captive elephants were sedated using a combination of medetomidine (9 μg/kg), butorphanol (30 μg/kg), and hyalase (2500 units) for standing sedation, with atipamezole (8 μg/kg) used as the antidote. The weights of the elephants were estimated by a qualified, experienced veterinarian with the help of elephant managers. Tranquilizer darts were shot from a vehicle using an airgun. Elephant handlers assisted the veterinarian with inserting microchips and ensuring that the elephants did not cause any harm to themselves or to people. The time from tranquilization to the first sign of reversal after administering the antidote ranged from 40 min to 2 h and 30 min.

In contrast, rehabilitated and wild elephants were tranquilized with etorphine (35 μg/kg), azaperone (400 μg/kg), and hyalase (5000 units), with naltrexone (35 μg/kg) administered as the antidote. These elephants were guided to accessible areas away from water to prevent drowning and darted from a helicopter. Once darted, a team of elephant handlers and researchers assisted the veterinarian in inserting microchips, fitting the collars, and keeping the elephants' trunks open to prevent suffocation. The average time from tranquilization to the first sign of reversal after administering the antidote was 15 min.

### 2.4 Release of Captive Elephants

A soft release approach was taken to minimize stress and allow acclimatization of captive elephants to life in the wild. The soft release occurred over three years, and different release phases were carried out sequentially with gradual changes to reduce human interaction. Initially, elephants were kept overnight in bomas at Abu Camp, consisting of small enclosures (approximately 1000 m^2^ for 4 to 5 elephants) fenced with electric wire, and were herded out each morning allowing them to forage and interact with wild elephants. They were monitored throughout the day by handlers until they were herded back to the Abu Camp bomas to overnight. Next, the elephants were walked to a new, larger boma (approximately 60,000 m^2^ for 4 to 5 elephants) located at the rewilding camp, 15 km south of the Abu Camp boma, where they continued with the same routine. Herding was gradually phased out until the boma gates were left open to allow free movement. Daily checks were conducted to monitor welfare; then all human interaction through physical monitoring ceased, and elephant positions were monitored remotely using the EarthRanger website (Seattle, Washington, USA), which allowed visualization of the GPS positions of the collared elephants. For analysis, all data collected for 499 days before the boma gates were left open (Before release) were compared to data collected for 597 days afterwards (After release), when captive elephant movements were no longer determined by humans. Therefore, rehabilitated and wild elephant data were also divided into Before and After release to allow for confounding environmental effects that could have impacted all elephants, and any social interactions between captive and rehabilitated elephants that used to be part of the same herd.

### 2.5 Movement Metrics

We selected metrics that would best represent the possible ways in which captive elephants may have altered their movement patterns when humans were not influencing them, considering sub‐hourly, daily, and seasonal movement metrics. Prior to release, captive elephants were herded during the day and returned to bomas at 18:00 for overnight confinement, which would have affected their movement metrics. After release, captive elephants could determine their own movement patterns in the same way as rehabilitated and wild elephants could throughout the study period.

#### 2.5.1 30‐min Distances

The 30‐min distances represented the distance between consecutive 30‐min GPS fixes for each elephant. 30‐min distances were categorized as either diurnal (06:00–17:59 h) or nocturnal (18:00–05:59 h), which corresponded to time spent in overnight bomas and daytime foraging hours for the captive elephants before release.

#### 2.5.2 Cumulative Daily Distance

The cumulative daily distance was calculated by summing all the distances between 30‐min GPS fixes in 24 h.

#### 2.5.3 Daily Displacement

Daily displacement was defined as the straight‐line distance between GPS fixes taken at 18:00 on consecutive days. This time corresponds to when captive elephants were returned to the boma each evening before release; therefore, any changes in displacement after release would directly indicate that the captive elephants deviated from the routine they were used to before release.

#### 2.5.4 Home Range Size

The monthly home range sizes of each collared elephant were calculated using kernel densities through the ‘kernelUD’ function in the adehabitatHR package (Calenge 2006).

### 2.6 Data Analysis

For all analyses, we used R v4.4.1 (R Core Team 2024). Given the seasonal changes in environmental conditions, we accounted for the interactive effect of season in all analyses. For each movement metric, we compared (i) captive elephants before and after release (Captive Before/After Release model): the movement metric was the dependent variable and the two‐way interaction between release phase (before/after) and season (early flood, late flood, and rainy) was the fixed effect; and captive, rehabilitated, and wild elephants. Then we compared between elephant groups (captive, rehabilitated, and wild elephant groups) (ii) before (Groups Before Release model), and (iii) after release (Groups After Release model): the movement metric was the dependent variable and the two‐way interaction between season and elephant group (captive, rehabilitated, or wild) was the fixed effect. For 30‐min distance analyses, we also included the effect of time of day to produce two three‐way interaction fixed effects (release phase, season and time of day; season, elephant group, and time of day). We used the ‘lme4’ package to fit generalized linear mixed models (GLMMs) with Gamma distributions and a log link function to model positive continuous responses and included a random intercept for individual elephants to account for repeated measures and individual variation (Bates 2010) (Equation 1). The most parsimonious models were identified using Akaike's Information Criterion corrected for small sample sizes (AICc) via the ‘dredge’ function in the ‘MuMin’ R package (Bartoń 2024). Pairwise comparisons of estimated marginal means were performed to identify significant differences using the “emmeans” R package (Lenth 2025).

(1) $$\begin{array}{l} Y_{\mathrm{ij}}\sim \text{Gamma}\left(\mu_{\mathrm{ij}},\varphi \right)\\ \gamma_{\mathrm{ij}}=\beta_{0}+\sum_{k=1}^{K}\beta_{k}X_{\mathrm{kij}}+b_{0j} \end{array}$$

where $Y_{\mathrm{ij}}$ is the response for observation $\left(i\right)$ of elephant $\left(j\right)$, $\mu_{\mathrm{ij}}$ is the expected value, φ is the dispersion parameter, $\gamma_{\mathrm{ij}}$ is the linear predictor, $\beta_{0}$ is the intercept, $\beta_{k}$ are coefficients for fixed effect predictors $X_{\mathrm{kij}}$ (main effects and interactions), and $b_{0j}\sim N\;\left(0,\sigma_{\text{Individual elephant}}^{2}\right)$ is the random intercept (Coelho et al. 2020).

## 3 Results

The total number of mean ± SD GPS fixes collected was 46,091 ± 6025, 45,372 ± 2321, and 34,975 ± 14,141 for captive, rehabilitated, and wild elephants, respectively. Eight elephants were collared for the full three‐year period (October 2021—November 2024). One wild elephant mistakenly had its collar removed after 18 months, so the collar was redeployed onto another wild elephant for the remaining 18 months. Two captive elephants died before the end of the project: one in November 2023 and the second in August 2024.

### 3.1 30‐Min Distances

The global models were the most parsimonious and included (i) a three‐way interaction among release phase, season and time of day for captive elephants (AIC = 3,288,798, AIC_ω_ = 1.00); (ii) a three‐way interaction among elephant group, season and time of day before release (AIC = 2,986,241, AIC_ω_ = 1.00); (iii) and a three‐way interaction among elephant group, season and time of day after release (AIC = 3,141,036, AIC_ω_ = 1.00); no other models were competitive.

**FIGURE 3:**
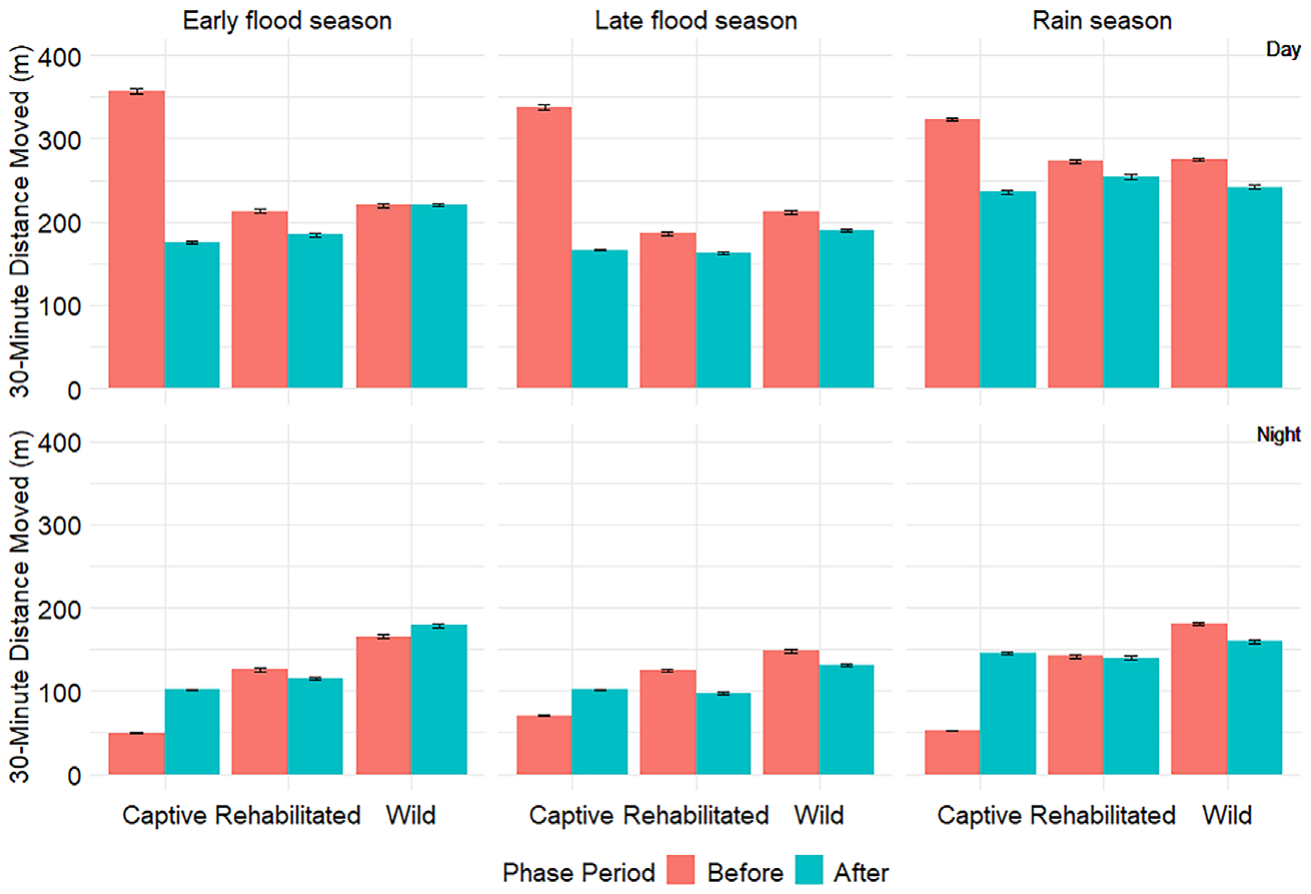
Seasonal mean daily displacement of African savannah elephants of different elephant groups collared in Abu Private Reserve, Okavango Delta, Botswana, before and after releasing captive elephants. The distance between the coordinates of GPS fixes recorded at 1800 h on consecutive days of an elephant was used to calculate the daily displacement.

In all seasons, captive elephants covered greater 30‐min distances during the day than at night before and after release (Figure 3, Table A1). Before release, 30‐min distances of captive elephants were smaller at night and greater during the day than those of rehabilitated and wild elephants (Figure 3, Table A1), although in the rainy season, the diurnal 30‐min distances of captive and rehabilitated elephants were not significantly different. After release, 30‐min distances of captive and rehabilitated elephants were not significantly different in any season (Figure 3, Table A1). Captive elephant 30‐min distances were significantly smaller than those of wild elephants in the day and night during the early flood season, and at night during the late flood season (Figure 3, Table A1). Diurnal 30‐min distances covered by rehabilitated and wild elephants did not differ significantly before or after release, but rehabilitated elephants had smaller nocturnal 30‐min distances than wild elephants during the early flood season before and after release, and during the late flood season after release (Figure 3, Table A1).

### 3.2 Cumulative Daily Distances

The global models were the most parsimonious and included (i) a two‐way interaction between release phase and season for captive elephants (AIC = 113378.5, AIC_ω_ = 1.00); (ii) a two‐way interaction between elephant group and season before release (AIC = 104602.6, AIC_ω_ = 1.00); (iii) and a two‐way interaction between elephant group and season after release (AIC = 109653.8, AIC_ω_ = 1.00); no other models were competitive.

**FIGURE 4:**
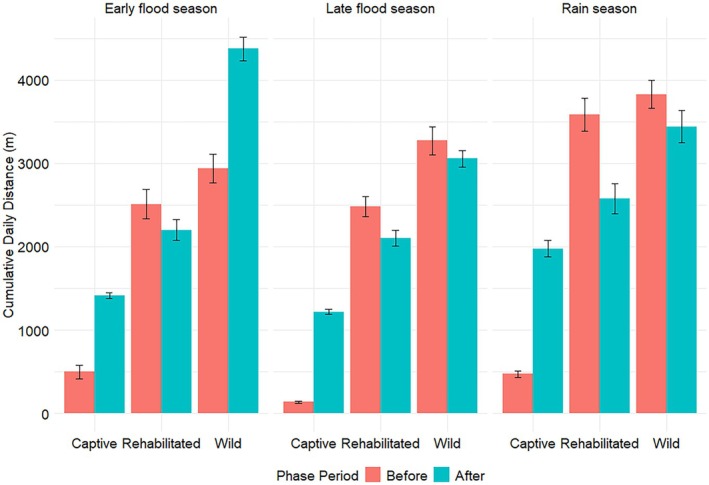
Seasonal mean 30‐min distances moved by African savannah elephants of different captivity levels divided by (top) diurnal and (bottom) nocturnal, of different elephant groups collared in Abu Private Reserve, Okavango Delta, Botswana, before and after releasing captive elephants. The diurnal movements were recorded from 0600 h to 1730 h, while the nocturnal movements were recorded from 1800 h to 0530 h. NB: The y axes of the two graphs are on different scales.

In all seasons, captive elephants covered greater cumulative daily distances (CDD) before than after release. Before release, CDDs of captive elephants were significantly greater than those of rehabilitated elephants during the early and late flood seasons but not during the rainy season (Figure 4, Table A2), and were the same as those of wild elephants in all seasons (Figure 4, Table A2). After release, CDDs of captive elephants were significantly lower than those of wild elephants during the early flood season but not during the late flood and rainy seasons, and were the same as those of rehabilitated elephants across all seasons. Within seasons and phases, there were no significant differences between CDDs of wild and rehabilitated elephants (Figure 4, Table A2).

### 3.3 Daily Displacement

The global models were the most parsimonious and included (i) a two‐way interaction between phase and season for captive elephants (AIC = 81532.0, AIC_ω_ = 1.00); (ii) a two‐way interaction between elephant group and season before release (AIC = 70499.1, AIC_ω_ = 1.00); (iii) and a two‐way interaction among elephant group and season after release (AIC = 84753.5, AIC_ω_ = 1.00); no other models were competitive.

**FIGURE 5:**
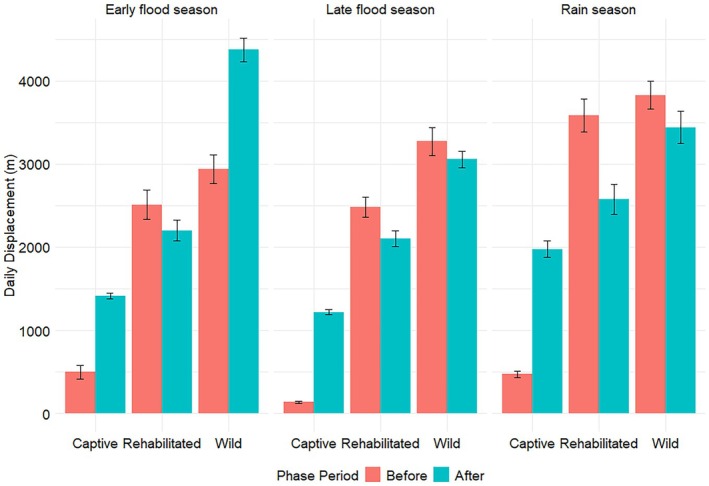
Seasonal mean daily ranges of African savannah elephants of different elephant groups collared in Abu Private Reserve, Okavango Delta, Botswana, before and after releasing captive elephants.

In all seasons, daily displacement (DD) of captive elephants was lower before than after release. Captive elephant DD was significantly lower than that of rehabilitated and wild elephants across all seasons before release (Figure 5, Table A3). After release, captive elephant DDs were significantly lower than those of rehabilitated elephants during the late flood season, and then those of wild elephants across all seasons (Figure 5, Table A3). Rehabilitated elephant DDs were not significantly different from those of wild elephants, except during the late flood season after release (Figure 5, Table A3).

### 3.4 Home Range Sizes

The global models were that the most parsimonious and included (i) a two‐way interaction between phase and season for captive elephants (AIC = 1889.9, AIC_ω_ = 1.00); (ii) a two‐way interaction between elephant group and season before release (AIC = 3482.0, AIC_ω_ = 1.00); (iii) and a two‐way interaction between elephant group and season after release (AIC = 810.6, AIC_ω_ = 1.00); no other models were competitive.

**FIGURE 6:**
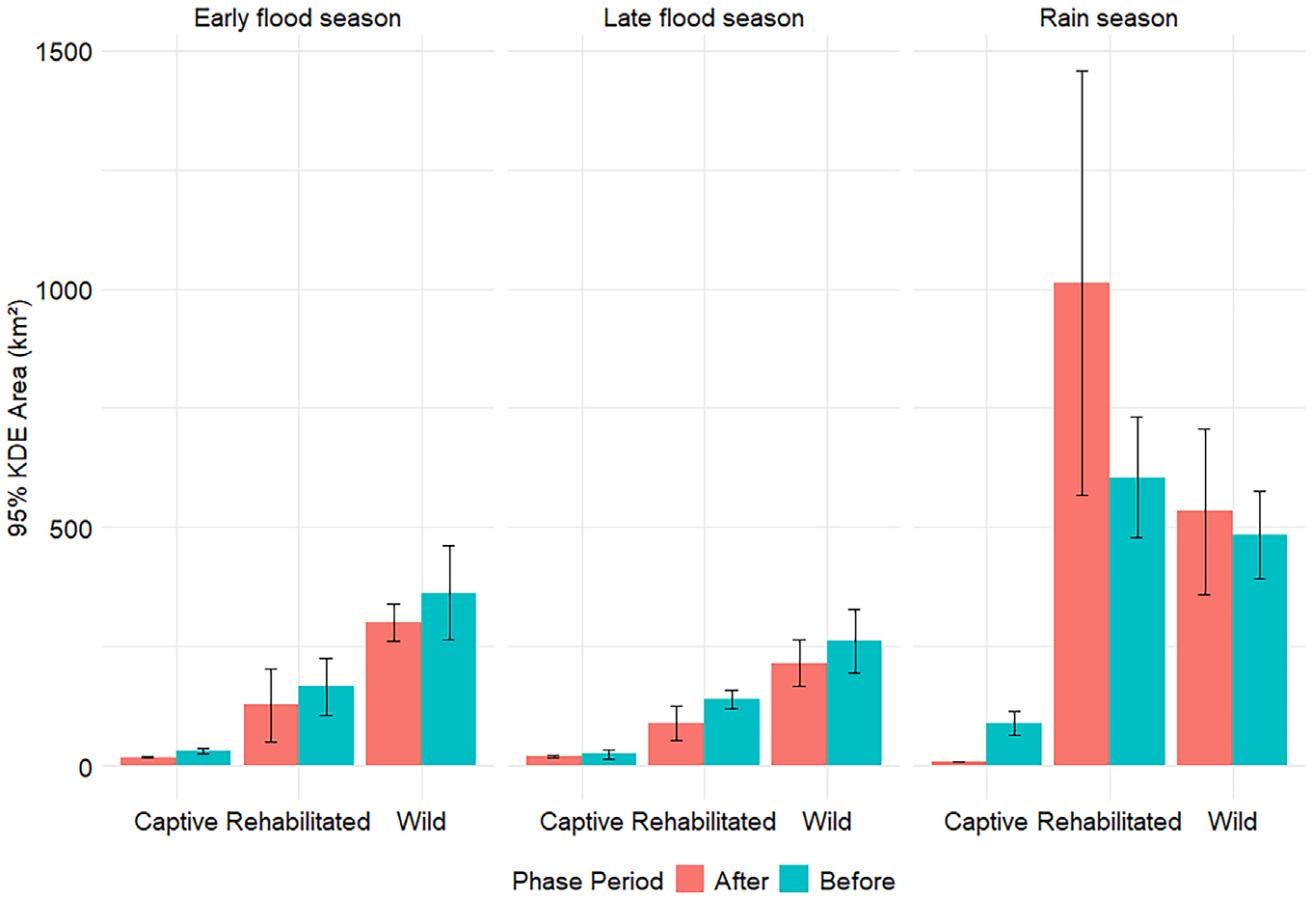
Seasonal mean monthly home range sizes (95% kernel density estimate) of African savannah elephants of different elephant groups collared in Abu Private Reserve, Okavango Delta, Botswana, before and after releasing captive elephants.

Captive elephant monthly home range (MHR) sizes did not vary before and after release, except during the rainy season, when their monthly home ranges were significantly smaller after release (Figure 6, Table A4). The MHRs of captive elephants were significantly smaller than those of rehabilitated and wild elephants across all seasons, both before and after release (Figure 6, Table A4). Rehabilitated and wild elephants occupied MHRs of the same size, except during the late flood season after release, when rehabilitated elephant MHRs were significantly smaller than those of wild counterparts (Figure 6, Table A4).

## 4 Discussion

Understanding the movement patterns of captive elephants during rewilding can bring insights into the rehabilitation and incorporation of those individuals into the wild. Captive elephants were released into the wild using a soft‐release approach over three years, and their movement patterns were compared to those of rehabilitated and wild elephants. Our results show mixed outcomes, with some hypotheses supported and others not. As predicted, captive elephant movement metrics changed after release, but this did not apply to the MHR sizes, which were not affected by the release. Captive elephants moved differently from rehabilitated and wild elephants before release, with significantly smaller nocturnal 30‐min distances and DDs (Tetzlaff et al. 2019; Doyle et al. 2024). After release, captive 30‐min distances (Figure 3, Table A1), CDDs (Figure 4, Table A2), and DDs (Figure 5, Table A3) during the early flood and rainy seasons were not significantly different from those of rehabilitated elephants, partially supporting the hypothesis that captive elephant movement metrics would mirror those of rehabilitated counterparts. However, MHR sizes (Figure 6, Table A4) remained consistently smaller, and CDDs were lower than those of wild elephants, meaning that captive elephant movement metrics still differed from those of wild conspecifics. These results align with Goldenberg et al. (2021), who used 1km^2^ grid cells to determine the movement metrics and space use of released captive elephants compared to wild elephants. Goldenberg et al. (2021) found that the captive elephants used fewer grid cells per given 14‐day period and visited fewer water points than wild elephants.

Seasonal effects on captive elephant movement metrics emerged only after release, indicating that human intervention before release may have constrained the capacity of captive elephants to alter their movements in response to changing environmental conditions (Loarie et al. 2009; Chamaillé‐Jammes et al. 2013). These patterns demonstrate the behavioral flexibility of elephants, in that release from captivity allowed elephants to gain some natural movement behaviors, and highlighted that captivity can leave a lasting effect, given that rehabilitated elephant movement metrics did not completely match those of wild elephants in the period of observation (Goldenberg et al. 2021). Similarly, captivity has been documented to have lasting effects across many different taxa: carnivores (Shier and Swaisgood 2012), ungulates (Morrison et al. 2021), and primates (Resende et al. 2021), with the behavior and movements of released individuals differing from those of wild conspecifics even years after release. In this study, the similarity between the movement metrics of captive and rehabilitated elephants may have stemmed from mutual learned behavior, social bonds, or shared risk‐avoidance strategies influenced by experiences in captivity (Evans et al. 2013a, 2013b; De Milliano et al. 2016).

Season affected the movement metrics of all elephants, except for captive elephants prior to release. This trend was also observed in captive elephants in South Africa (Roos et al. 2024), suggesting similar management strategies by humans in different seasons during captivity. The absence of seasonal effects on captive elephant movement metrics before release underscores the impact of management, whereby confinement to bomas at night and daily herding masked natural movement patterns (Doyle et al. 2024; Roos et al. 2024). As predicted, elephant movement metrics were greatest during the rainy season, reflecting the wide distribution of available surface water and forage (Birkett et al. 2012; Makati et al. 2025). Lower movement metric values during the early flood and late flood seasons suggest a dependence on permanent water sources that are less widely distributed, a pattern that is consistent with the behavior of other large herbivores such as blue wildebeest (Boone et al. 2006) and Cape buffalo (Naidoo et al. 2012).

Contrary to our prediction, captive elephants covered greater CDDs before than after release. Captive elephants were actively herded within an approximate 5 km radius around the overnight boma, which meant that the minimum CDD would have been 10 km, which appears to be larger than that of other elephants. The cessation of these human‐guided movements after release means that the captive elephants may have then optimized their movements to more energy‐efficient patterns (Ross 2006; Egevang et al. 2010). Some of the movement metrics of rehabilitated elephants differed from their wild counterparts, but their survival for over a decade in the wild indicates that such differences do not lead to greater risk of mortality (Beirne et al. 2021), especially since elephants are not largely vulnerable to predation in the wild like other species (Domínguez‐Rodrigo and Baquedano 2025). This underscores the importance of realistic expectations that released elephants may never entirely mirror wild elephants but can still survive, reproduce, and contribute ecologically, which may be sufficient for conservation goals (Goldenberg et al. 2021). However, the movement patterns could have been influenced by the small sample sizes used in the study, which could have led to high variation (Delsink et al. 2013; Beirne et al. 2021; Tiller et al. 2022). Nevertheless, captive elephants can be expected to survive effectively in the wild after release.

In conclusion, the movement metrics of captive elephants were significantly affected by the release, showing partial matching with movement metrics of rehabilitated and wild conspecifics, though differences persisted in MHR sizes and CDDs. Seasonal patterns emerged only after release, highlighting adjustment to seasonal cues in the environment once human‐induced constraints were lifted. These results emphasize that while rewilding allows elephants to regain many natural behaviors, captivity likely leaves lasting effects that may prevent full behavioral convergence with wild elephants. Therefore, conservation programs should adopt long‐term monitoring and realistic expectations for rewilding outcomes, recognizing that “success” may lie not in complete convergence but in the ability of the released animals to persist and function in the environment they are released in.

## Author Contributions

**Murphy Tladi:** conceptualization (lead), data curation (lead), formal analysis (lead), investigation (lead), methodology (lead), validation (lead), visualization (lead), writing – original draft (lead), writing – review and editing (lead). **Mike Murray‐Hudson:** conceptualization (supporting), data curation (supporting), formal analysis (supporting), investigation (supporting), methodology (supporting), project administration (supporting), supervision (supporting), validation (equal), writing – review and editing (equal). **Andre Ganswindt:** conceptualization (supporting), data curation (supporting), formal analysis (supporting), investigation (supporting), methodology (supporting), supervision (supporting), validation (supporting), visualization (supporting), writing – review and editing (equal). **Emily Bennitt:** conceptualization (equal), data curation (equal), formal analysis (supporting), funding acquisition (lead), investigation (equal), methodology (equal), project administration (lead), resources (lead), supervision (lead), validation (supporting), visualization (supporting), writing – original draft (equal), writing – review and editing (equal).

## Funding

This work was supported by Abu Private Reserve.

## Conflicts of Interest

The authors declare no conflicts of interest.

## Data Availability

The data and the R scripts (Tladi et al. 2025) used for analysis in this paper are available on the Dryad Digital Repository website DOI (https://doi.org/10.5061/dryad.6hdr7src3).

## Appendix A

**TABLE A1:** Pairwise comparisons of fixed variables affecting 30‐min distances of captive, rehabilitated, and wild elephants in Abu Private Reserve, Okavango Delta, Botswana, across different times of day, before and after release, and seasons.

Elephants	Phase	Time of day	Season	Estimate	SE	*z*. ratio	*p*
Captive	Before Vs After	Day	Early flood	0.747	0.01	74.574	< 0.0001*
Captive	Before Vs After	Day	Late flood	0.731	0.0098	74.642	< 0.0001*
Captive	Before Vs After	Day	Rain	0.356	0.0110	32.287	< 0.0001*
Captive	Before Vs After	Night	Early flood	−0.698	0.0103	−67.704	< 0.0001*
Captive	Before Vs After	Night	Late flood	−0.338	0.0101	−33.417	< 0.0001*
Captive	Before Vs After	Night	Rain	−0.974	0.0113	−86.098	< 0.0001*
Captive vs. Rehabilitated	Before	Day	Early flood	0.519698	0.0800	6.497	< 0.0001*
Captive vs. Rehabilitated	Before	Day	Late flood	0.594087	0.0798	7.443	< 0.0001*
Captive vs. Rehabilitated	Before	Day	Rain	0.170989	0.0793	2.157	0.0787
Captive vs. Wild	Before	Day	Early flood	0.502242	0.0689	7.287	< 0.0001*
Captive vs. Wild	Before	Day	Late flood	0.455320	0.0690	6.600	< 0.0001*
Captive vs. Wild	Before	Day	Rain	0.170146	0.0683	2.490	0.0341*
Rehabilitated vs. Wild	Before	Day	Early flood	−0.017456	0.0898	−0.194	0.9794
Rehabilitated vs. Wild	Before	Day	Late flood	−0.138767	0.0897	−1.547	0.2691
Rehabilitated vs. Wild	Before	Day	Rain	−0.000843	0.0890	−0.009	1
Captive vs. Rehabilitated	Before	Night	Early flood	−0.937064	0.0800	−11.717	< 0.0001*
Captive vs. Rehabilitated	Before	Night	Late flood	−0.566753	0.0799	−7.097	< 0.0001*
Captive vs. Rehabilitated	Before	Night	Rain	−0.998132	0.0793	−12.594	< 0.0001*
Captive vs. Wild	Before	Night	Early flood	−1.195649	0.0690	−17.340	< 0.0001*
Captive vs. Wild	Before	Night	Late flood	−0.738194	0.0691	−10.688	< 0.0001*
Captive vs. Wild	Before	Night	Rain	−1.237024	0.0684	−18.097	< 0.0001*
Rehabilitated vs. Wild	Before	Night	Early flood	−0.258585	0.0898	−2.881	0.0111*
Rehabilitated vs. Wild	Before	Night	Late flood	−0.171441	0.0898	−1.910	0.1359
Rehabilitated vs. Wild	Before	Night	Rain	−0.238892	0.0889	−2.686	0.0198*
Captive vs. Rehabilitated	After	Day	Early flood	−0.09135	0.145	−0.628	0.8048
Captive vs. Rehabilitated	After	Day	Late flood	−0.02029	0.145	−0.139	0.9893
Captive vs. Rehabilitated	After	Day	Rain	−0.15066	0.146	−1.031	0.5573
Captive vs. Wild	After	Day	Early flood	−0.30532	0.110	−2.769	0.0155*
Captive vs. Wild	After	Day	Late flood	−0.21361	0.110	−1.937	0.1284
Captive vs. Wild	After	Day	Rain	−0.13645	0.111	−1.231	0.4348
Rehabilitated vs. Wild	After	Day	Early flood	−0.21397	0.155	−1.379	0.3518
Rehabilitated vs. Wild	After	Day	Late flood	−0.19332	0.155	−1.246	0.426
Rehabilitated vs. Wild	After	Day	Rain	0.01422	0.156	0.091	0.9954
Captive vs. Rehabilitated	After	Night	Early flood	−0.16043	0.145	−1.103	0.5124
Captive vs. Rehabilitated	After	Night	Late flood	0.00134	0.145	0.009	1
Captive vs. Rehabilitated	After	Night	Rain	−0.05620	0.146	−0.385	0.9217
Captive vs. Wild	After	Night	Early flood	−0.62084	0.110	−5.637	< 0.0001*
Captive vs. Wild	After	Night	Late flood	−0.32654	0.110	−2.960	0.0086*
Captive vs. Wild	After	Night	Rain	−0.21749	0.111	−1.961	0.1221
Rehabilitated vs. Wild	After	Night	Early flood	0.46041	0.155	−2.968	0.0084*
Rehabilitated vs. Wild	After	Night	Late flood	−0.32789	0.155	−2.113	0.0872
Rehabilitated vs. Wild	After	Night	Rain	−0.16129	0.156	−1.035	0.5547

**TABLE A2:** Pairwise comparisons of fixed variables affecting cumulative daily distances of captive, rehabilitated, and wild elephants in Abu Private Reserve, Okavango Delta, Botswana, before and after release across different seasons.

Elephants	Phase	Season	Estimate	SE	*z*. ratio	*p*
Captive	Before Vs After	Early flood	0.4335	0.0123	35.171	< 0.0001*
Captive	Before Vs After	Late flood	0.4525	0.0120	37.701	< 0.0001*
Captive	Before Vs After	Rain	0.0683	0.0133	5.127	< 0.0001*
Captive vs. Rehabilitated	Before	Early flood	0.3025	0.0906	3.340	0.0024*
Captive vs. Rehabilitated	Before	Late flood	0.3651	0.0903	4.042	0.0002*
Captive vs. Rehabilitated	Before	Rain	0.0408	0.0896	0.456	0.8919
Captive vs. Wild	Before	Early flood	0.1216	0.0778	1.563	0.2618
Captive vs. Wild	Before	Late flood	0.1446	0.0778	0.1858	0.151
Captive vs. Wild	Before	Rain	−0.1406	0.0770	−1.827	0.1609
Rehabilitated vs. Wild	Before	Early flood	−0.1809	0.1010	−1.796	0.171
Rehabilitated vs. Wild	Before	Late flood	−0.2205	0.1010	−2.190	0.0729
Rehabilitated vs. Wild	Before	Rain	−0.1814	0.0997	−1.819	0.1632
Captive vs. Rehabilitated	After	Early flood	−0.04516	0.183	−0.246	0.9671
Captive vs. Rehabilitated	After	Late flood	0.02966	0.183	0.162	0.9857
Captive vs. Rehabilitated	After	Rain	0.00218	0.184	0.012	0.9999
Captive vs. Wild	After	Early flood	−0.44668	0.134	−3.338	0.0024*
Captive vs. Wild	After	Late flood	−0.28694	0.134	−2.137	0.0826
Captive vs. Wild	After	Rain	−0.21931	0.135	−1.623	0.236
Rehabilitated vs. Wild	After	Early flood	−0.40152	0.188	−2.137	0.0825
Rehabilitated vs. Wild	After	Late flood	−0.31661	0.188	−1.683	0.2118
Rehabilitated vs. Wild	After	Rain	−0.22150	0.189	−1.172	0.4702

**TABLE A3:** Pairwise comparisons of fixed variables affecting the daily displacement of captive, rehabilitated, and wild elephants in Abu Private Reserve, Okavango Delta, Botswana, before and after release across different seasons.

Elephants	Phase	Season	Estimate	SE	*z*. ratio	*p*
Captive	Before Vs After	Early flood	−0.995	0.0574	−17.344	< 0.0001*
Captive	Before Vs After	Late flood	−2.179	0.0573	−38.013	< 0.0001*
Captive	Before Vs After	Rain	−1.379	0.0659	−20.916	< 0.0001*
Captive vs. Rehabilitated	Before	Early flood	−1.5837	0.174	−9.111	< 0.0001*
Captive vs. Rehabilitated	Before	Late flood	−2.9302	0.166	−17.610	< 0.0001*
Captive vs. Rehabilitated	Before	Rain	−2.0225	0.162	−12.508	< 0.0001*
Captive vs. Wild	Before	Early flood	−1.7681	0.145	−12.189	< 0.0001*
Captive vs. Wild	Before	Late flood	−3.2027	0.145	−22.141	< 0.0001*
Captive vs. Wild	Before	Rain	−2.0717	0.135	−15.349	< 0.0001*
Rehabilitated vs. Wild	Before	Early flood	−0.1845	0.194	−0.950	0.6087
Rehabilitated vs. Wild	Before	Late flood	−0.2725	0.188	−1.451	0.3148
Rehabilitated vs. Wild	Before	Rain	−0.0493	0.180	−0.274	0.9595
Captive vs. Rehabilitated	After	Early flood	−0.482	0.210	−2.301	0.0556
Captive vs. Rehabilitated	After	Late flood	−0.587	0.209	−2.805	0.0139*
Captive vs. Rehabilitated	After	Rain	−0.372	0.218	−1.711	0.2009
Captive vs. Wild	After	Early flood	−1.137	0.172	−6.617	< 0.0001*
Captive vs. Wild	After	Late flood	−0.928	0.173	−5.381	< 0.0001*
Captive vs. Wild	After	Rain	−0.600	0.179	−3.359	0.0023*
Rehabilitated vs. Wild	After	Early flood	−0.654	0.227	−2.878	0.0111*
Rehabilitated vs. Wild	After	Late flood	−0.342	0.227	−1.503	0.2895
Rehabilitated vs. Wild	After	Rain	−0.227	0.236	−0.963	0.6002

**TABLE A4:** Pairwise comparisons of fixed variables affecting monthly home ranges of captive, rehabilitated, and wild elephants in Abu Private Reserve, Okavango Delta, Botswana, before and after release across different seasons.

Elephants	Phase	Season	Estimate	SE	*z*. ratio	*p*
Captive	Before Vs After	Early flood	0.02324	0.0195	1.189	0.2344
Captive	Before Vs After	Late flood	0.00766	0.0135	0.569	0.5694
Captive	Before Vs After	Rain	0.09555	0.0331	2.887	0.0039*
Captive vs. Rehabilitated	Before	Early flood	−1.661	0.425	−3.904	0.0003*
Captive vs. Rehabilitated	Before	Late flood	−1.900	0.434	−4.380	< 0.0001*
Captive vs. Rehabilitated	Before	Rain	−1.947	0.426	−4.566	< 0.0001*
Captive vs. Wild	Before	Early flood	−2.348	0.351	−6.684	< 0.0001*
Captive vs. Wild	Before	Late flood	−2.433	0.364	−6.686	< 0.0001*
Captive vs. Wild	Before	Rain	−1.454	0.354	−4.107	0.0001*
Rehabilitated vs. Wild	Before	Early flood	−0.687	0.462	−1.488	0.2964
Rehabilitated vs. Wild	Before	Late flood	−0.533	0.474	−1.125	0.4986
Rehabilitated vs. Wild	Before	Rain	0.493	0.462	1.068	0.5343
Captive vs. Rehabilitated	After	Early flood	−1.875	0.480	−3.907	0.0003*
Captive vs. Rehabilitated	After	Late flood	−1.071	0.421	−2.548	0.0292*
Captive vs. Rehabilitated	After	Rain	−4.468	0.470	−9.504	< 0.0001*
Captive vs. Wild	After	Early flood	−2.931	0.415	−7.054	< 0.0001*
Captive vs. Wild	After	Late flood	−2.424	0.352	−6.888	< 0.0001*
Captive vs. Wild	After	Rain	−3.949	0.404	−9.770	< 0.0001*
Rehabilitated vs. Wild	After	Early flood	−1.056	0.522	−2.022	0.107
Rehabilitated vs. Wild	After	Late flood	−1.353	0.453	−2.986	0.008*
Rehabilitated vs. Wild	After	Rain	0.519	0.524	0.991	0.5828
