# Correction to “Release From Captivity Allows African Savannah Elephant Movement Patterns to Converge With Those of Wild and Rehabilitated Conspecifics”

**DOI:** 10.1002/ece3.72993

**Published:** 2026-01-27

Tladi, M., M. Murray‐Hudson, A. Ganswindt, and E. Bennitt. 2025. “Release From Captivity Allows African Savannah Elephant Movement Patterns to Converge With Those of Wild and Rehabilitated Conspecifics.” *Ecology and Evolution* 15, no. 12: e72597. https://doi.org/10.1002/ece3.72597.

In the results section:

1. Caption of Figure 3 text “Seasonal mean daily displacement of African savannah elephants of different elephant groups collared in Abu Private Reserve, Okavango Delta, Botswana, before and after releasing captive elephants. The distance between the coordinates of GPS fixes recorded at 1800 h on consecutive days of an elephant was used to calculate the daily displacement” is incorrect and should read “Seasonal mean 30‐min distances moved by African savannah elephants of different elephant groups divided by (top) diurnal and (bottom) nocturnal, collared in Abu Private Reserve, Okavango Delta, Botswana, before and after releasing captive elephants. The diurnal movements were recorded from 0600 h to 1730 h, while the nocturnal movements were recorded from 1800 h to 0530 h. NB: The y axes of the two graphs are on different scales.”

2. Figure 4 is incorrect. The correct figure is displayed below:

**FIGURE 4:**
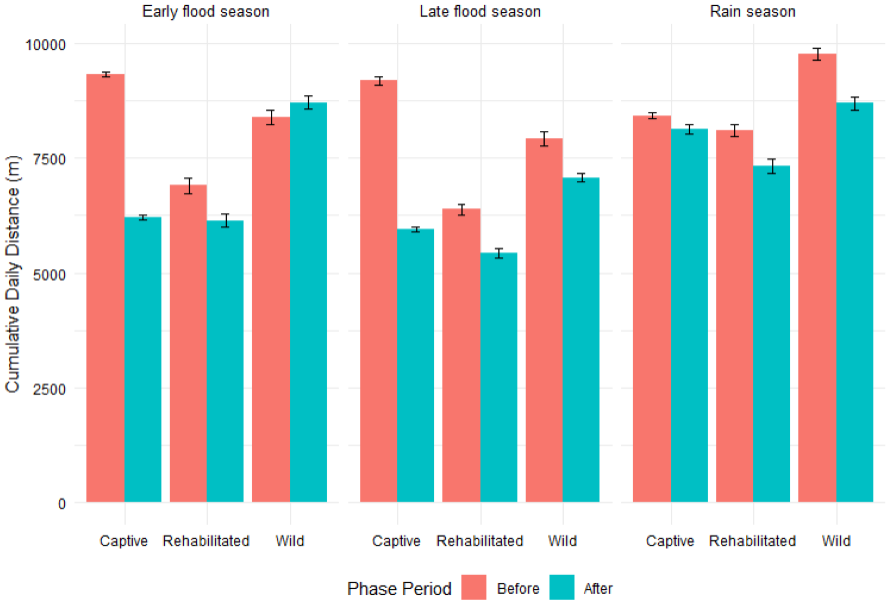
Seasonal mean cumulative daily distances of African savannah elephants of different elephant groups collared in Abu Private Reserve, Okavango Delta, Botswana, before and after releasing captive elephants.

3. Caption of Figure 4 text “Seasonal mean 30‐min distances moved by African savannah elephants of different captivity levels divided by (top) diurnal and (bottom) nocturnal, of different elephant groups collared in Abu Private Reserve, Okavango Delta, Botswana, before and after releasing captive elephants. The diurnal movements were recorded from 0600 h to 1730 h, while the nocturnal movements were recorded from 1800 h to 0530 h. NB: The y axes of the two graphs are on different scales.” is incorrect and should read “Seasonal mean cumulative daily distances moved by African savannah elephants of different elephant groups collared in Abu Private Reserve, Okavango Delta, Botswana, before and after releasing captive elephants.”

4. Caption of Figure 5 text “Seasonal mean daily ranges of African savannah elephants of different elephant groups collared in Abu Private Reserve, Okavango Delta, Botswana, before and after releasing captive elephants.” is incorrect and should read “Seasonal mean daily displacement of African savannah elephants of different elephant groups collared in Abu Private Reserve, Okavango Delta, Botswana, before and after releasing captive elephants. The distance between the coordinates of GPS fixes recorded at 1800 h on consecutive days of an elephant was used to calculate the daily displacement.”

5. In the references section, reference “Tladi, M., M. Murray‐Hudson, A. Ganswindt, and E. Bennitt. 2025. Release From Captivity Allows African Savannah Elephant Movement Patterns to Converge With Those of Wild and Rehabilitated Conspecifics. 1st ed. Dryad Digital Repository.” is not correct and should read “Tladi, M., M. Murray‐Hudson, A. Ganswindt, and E. Bennitt. 2025. Data from: Release From Captivity Allows African Savannah Elephant Movement Patterns to Converge With Those of Wild and Rehabilitated Conspecifics. 1st ed. Dryad Digital Repository.”

The online version of this article has been corrected accordingly.

We apologize for these errors.

